# Experimental Study on Vibratory Compaction Behavior of Tunneling Rock Wastes Used as Unbound Permeable Aggregate Base Materials

**DOI:** 10.3390/ma15228016

**Published:** 2022-11-14

**Authors:** Yuliang Chen, Qunding Yu, Wenqi Li, Yuanjie Xiao, Tao Yang, Zhiyong Li, Xiao Zhi, Pin Deng

**Affiliations:** 1 Hunan Communications Research Institute Co., Ltd., Changsha 410015, China; 2 Urban Rail and Underground Engineering Design and Research Institute, China Railway Siyuan Survey and Design Group Co., Ltd., Wuhan 430063, China; 3 Department of Geotechnical Engineering, School of Civil Engineering, Central South University, Changsha 410075, China; 4 MOE Key Laboratory of Engineering Structures of Heavy Haul Railway (Central South University), Changsha 410075, China; 5 Department of Research & Development, China Building Materials Research Institute, Beijing 100024, China

**Keywords:** tunneling rock wastes, unbound permeable aggregate base, plate vibratory compaction, degree of saturation, normalized compaction curves, particle breakage

## Abstract

The tunneling rock wastes (TRW) have been increasingly generated and stockpiled in massive quantities. Recycling them for use as unbound granular pavement base/subbase materials has become an alternative featuring low carbon emission and sustainability. However, the field compaction of such large-size, open-graded materials remains challenging, thus affecting post-construction deformation and long-term stability of such pavement base/subbase layers. This study conducted a series of proctor compaction and new plate vibratory compaction tests to analyze the compaction characteristics of such TRW materials. A total of six different open gradations were designed from particle packing theory. In addition, the effects of gradation and compaction methods on the compaction characteristics, particle breakage of TRW materials, and the optimal combination of vibratory parameters were investigated by normalizing the curves of achieved dry density versus degree of saturation for various combinations of gradations, compaction methods, and compaction energy levels. The post-compaction characteristics of interparticle contact, pore structure, and particle breakage were analyzed from the X-ray computed topography (XCT) scanning results of TRW specimens with different gradations. The findings showed that the gravel-to-sand ratio (*G*/*S*) based gradation design method can effectively differentiate distinct types of particle packing structures. There exists an optimal *G*/*S* range that could potentially result in the highest maximum dry density, the lowest particle breakage, and the best pore structure of compacted unbound permeable aggregate base (UPAB) materials. The achieved dry density (ρ*_d_*) of UPAB materials subjected to vibratory plate compaction exhibited three distinct phases with compaction time, from which the optimal excitation frequency range was found to be 25–27 Hz and the optimal combination of vibratory parameters were determined. The normalized compaction curves of degree of saturation versus achieved dry density were found insensitive to changes in material gradations, compaction methods and energy levels, thus allowing for a more accurate evaluation and control of field compaction quality.

## 1. Introduction

The permeable pavement structure layer has many advantages over the conventional impervious pavement structure, including a higher void ratio, greater hydraulic conductivity, better water permeability, anti-skid, sound absorption, and noise reduction [1,2]. Therefore, the partial or complete use of a permeable pavement structure layer is an effective method to enhance the drainage and ecological functions of the road system [3], which is also initial to the construction of Sponge City. Field compaction is one of the most critical aspects in the paving process of pavement structural layers. Different types of external compaction forces (such as static or dynamic load with varying frequencies and amplitudes) are applied to the structural layer of the materials so that the filler particles move relatively to attain the dense state [4]. Compaction degree is an important performance index of pavement quality, and the improvement of compaction quality can enhance the compactness (void volume, size, spatial distribution, etc.) of the pavement structure layer, thereby increasing its service performance such as permanent deformation resistance and durability [5]. While the field compaction roller and its compaction parameters determine the compaction quality, it is the most crucial factor in achieving the compaction standard [6]. Most existing research focuses on the compaction performance of pavement surfaces, while relatively little is known about the compaction efficiency of unbound aggregate materials used in flexible base and subbase, especially unbound permeable aggregate base (UPAB) materials. Traditional dense graded or new unbound permeable aggregate base materials used in pavement base structure mainly have three functions: (1) providing uniform, stable and durable support for the surface layer, (2) transmitting and distributing the traffic load acting on the base layer, and (3) providing adequate water drainage (permeability) capacity. Insufficient compaction degree and the high void ratio will lead to insufficient bearing capacity and deformation stability of UPAB and induce failure and instability such as unstable failure and excessive permanent deformation of permeable pavement structure [7]. Therefore, it is vital to investigate the compaction characteristics and the influencing factors of UPAB materials in depth to improve the service performance of permeable base layer under repeated traffic loads and expand the scope of its engineering application.

Researchers have utilized a variety of compaction methods to prepare laboratory specimens of recycled aggregates and studied engineering characteristics of field-compacted pavement structures through laboratory compaction tests [8,9,10,11,12,13]. The vibration compaction technique has become a widespread approach for pavement compaction due to its high efficiency, ability to compact large thickness, and accurate simulation of the impact of field vibration rolling on the pavement structural layer of the materials [5]. Feng and Liang [14,15] investigated the influence of graded macadam distribution, vibration frequency, action time, and excitation force on the compaction characteristics during laboratory surface vibration compaction. They determined an optimal frequency, action time, and excitation force to obtain the maximum dry density of the compacted layer. Wersäll et al. [16] analyzed the effect of frequency of vibratory roller on compaction effect in field tests and discovered that lower working frequency can decrease energy consumption without reducing the compaction effect. Furthermore, the optimal vibratory compaction frequency of compacted soil is slightly greater than the natural frequency of soil. Mo and Li [17,18] optimized the vibration parameters and time of the vertical vibrator, which can better simulate the field compaction conditions. Yingjun [19] studied the vibration compactor and its selection standard for lime-fly ash macadam and proposed the optimal vibration frequency and time of this material. JinGang [20] compared the difference in shear strength of graded crushed stone samples obtained by manual layered compaction and vibration molding with the same level of compaction. Jian [21] employed a surface vibration meter to compare the gradation changes of cement stabilized macadam before and after vibration and static pressure molding. Guanghui [22] analyzed the interaction between unbound aggregate base materials and vibration compactor and proposed to judge the compaction quality according to the change in structural resistance of unbound aggregate base materials, thereby altering the previous evaluation system only using dry density index. On this basis, Guangjie [23] proposed that the change of acceleration response of vibratory compactor should be utilized to characterize the compaction process, which is beneficial for distinguishing the compaction state on-site. Based on the influence law of rolling parameters of a vibratory roller on vibration measurement, Erlu [24] established the relationship between them and formed a highly applicable method for evaluating the quality of continuous compaction control. Qinglong [25] developed a compaction analyzer using an artificial neural network (ANN) and a unique assumption of vibration characteristics of asphalt mixture by vibration compactor, which can be utilized to predict the compaction density of asphalt mixture continuously. Based on the real-time dynamic global positioning system (GPS) and road roller’s integrated acoustic detection technology, the sonic compaction value (SCV) was adopted as the real-time monitoring index of rockfill compaction quality and a method for evaluating rockfill compaction quality based on SCV. Nagaraja [26] predicted the maximum dry density and the optimal water content after compaction using the plastic limit of soil after vibratory compaction and established a corresponding good relationship. Kavussi [27] studied the influence of moisture content and subgrade resilience modulus on the compaction level of unbound aggregate materials and found a significant linear relationship between resilience modulus and compaction level of unbound aggregate base materials. Jidou [28] employed the surface vibration compaction method to compact the discontinuously graded coarse-grained soil and built the prediction model. Erlu [24] investigated the influence of gradation on the dry density of compacted coarse-grained soil utilizing a surface vibration compactor and proposed the optimal gradation interval of dry density.

The above studies on vibratory compaction of unbound aggregate base materials focus on the parameter optimization of compaction instruments and comparing different compaction methods. However, they fail to effectively unify the compaction effects of different compaction methods and consider the coupling effect between roller and subgrade soil, resulting in a significant disparity between the laboratory compaction test findings and the actual field compaction quality. In addition, there is much research on the compaction characteristics and response of coarse-grained soil materials but little on the compaction characteristics of UPAB materials. This paper compares a new laboratory plate vibratory compaction test to the traditional proctor compaction based on various graded unbound permeable aggregate base materials. Multiple aspects of the influences of different compaction methods, vibration frequency, vibration force, and vibration time on the compaction characteristics and effects of different UPAB fillers are comprehensively analyzed. The vibration parameter combination with the best compaction effect and the optimal design method of permeable grading are prompted. It can provide theoretical and technical guidance for the field compaction construction of permeable graded gravel foundations and intelligent compaction technology.

## 2. Laboratory Test

### 2.1. Aggregate Properties

**Figure 1:**
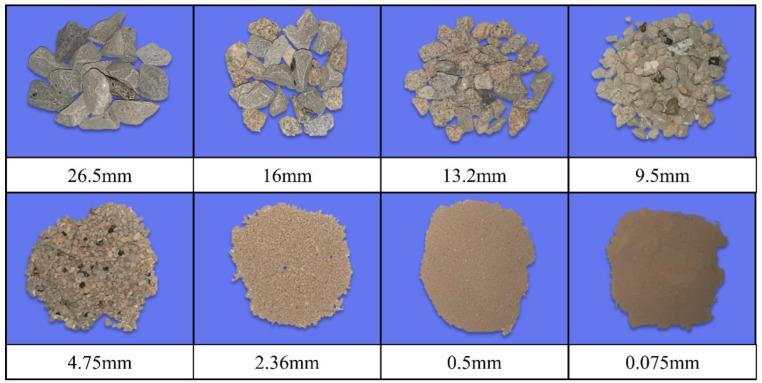
Illustration of aggregate materials within the different size ranges from sieve analysis.

The unbound permeable aggregate base (UPAB) materials used in this study were sampled from the tunnelling rock wastes stockpiled in a nearby quarry in the suburb of Changsha city, China. The UPAB materials derived from tunnelling rock wastes in this study refer to those that are open-graded in nature and relatively more permeable than conventional dense-graded aggregate base materials derived from natural stones. In addition, the former is more angular and rougher in terms of particle morphology than the latter. The UPAB materials are more suitable for mitigating flooding problems and promoting sponge city initiatives than conventional aggregate base materials. In addition, the UPAB materials used in this study were sampled from tunneling rock wastes, thus resulting in additional benefits including lower construction cost, reduced carbon emission, and greater sustainability and environmental friendliness. Figure 1 indicates that the aggregate with a minimum particle size of 2.36 mm was washed, dried, and then divided into five categories: 26.5 mm, 16 mm, 13.2 mm, 9.5 mm, and 4.75 mm. The fine particle less than 2.36 mm was sieved in three particle sizes: 2.36 mm, 0.5 mm, and 0.075 mm.

For the laboratory compaction test, the gradation of the UPAB sample was designed and controlled by the stone-sand ratio (*G*/*S*) index suggested by the author in the previous study [29]. The *G*/*S* concept and its calculation were originally a method proposed by Sánchez-Leal [30] for the gradation design of asphalt mixture based on the Talbot function. The study was extended to the gradation design of UPAB by verifying the mechanical performance of different *G*/*S* levels of graded crushed stone packing. It discovered that the *G*/*S* value has well mechanical performance indicators such as the shear strength and accumulative plastic deformation of graded crushed stone. Specifically, the *G*/*S* parameter can be determined from the maximum particle size of the particles and the shape parameter of the gradation curve using Equation (1), where the shape parameter of the gradation curve can be identified by the Talbot function (Equation (2)).

(1) $$G/S=\frac{1-P_{4.75}}{1-(1-P_{4.75})-P_{0.075}}=\frac{D_{\max}^{n}-4.75^{n}}{4.75^{n}-0.075^{n}},$$

(2) $$P_{i}={\left(\frac{d_{i}}{D_{\max}}\right)}^{n},$$

where *P_i_* is the *i*-th cumulative sieving rate, *d_i_* is the diameter of the *i*-th sieve (mm), *D*_max_ is the maximum particle size (mm), and *n* is the gradation shape parameter.

**Figure 2:**
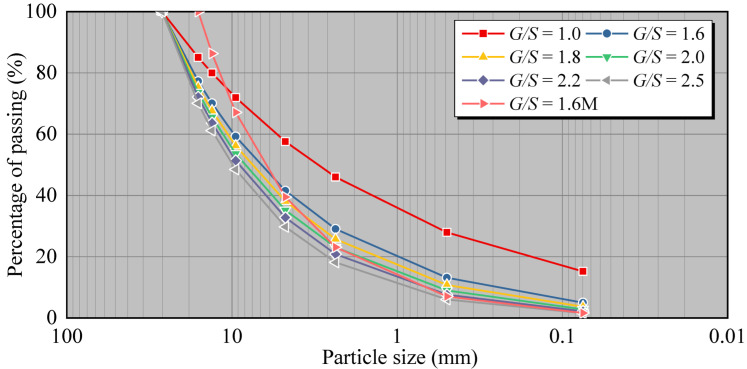
Gradation Curves of UPAB Specimens Used for Laboratory Compaction Tests.

The maximum particle size of graded gravel used for pavement bases or subbases of various traffic load levels should not exceed 26.5 mm, and the percentage of particles passing through a 0.075 mm sieve should not surpass 15% [31]. According to the principle of boundary particle size-volume filling of graded crushed stone, Chen Jian [21] proposed dividing the gradation in the specification into three ranges: upper, median, and lower limit, corresponding to three different types of packing structures of filler particles—skeleton void, dense skeleton, and dense suspension. Since diverse packing structures of filler particles can lead to significant differences in mechanical and engineering performance, comprehensively considering the relevant specifications of the graded gravel bed structure and the design and construction of the highway pavement structure, the maximum particle size of the gravel filler is selected to be 26.5 mm, and the crushing is controlled. The *G*/*S* values of the stone gradation are 1.0, 1.6, 1.8, 2.0, 2.2, and 2.5, respectively. To examine the impact of the maximum particle size, one group of gradations has a maximum particle size of 16 mm, and the *G*/*S* value of 1.6 (referred to as 1.6 M in the text). The gradation design scheme and related parameters of the compaction test are listed in Table 1, and the gradation curve of each grade of crushed stone filler sample is depicted in Figure 2.

### 2.2. New Plate Vibratory Compaction Apparatus

**Figure 3:**
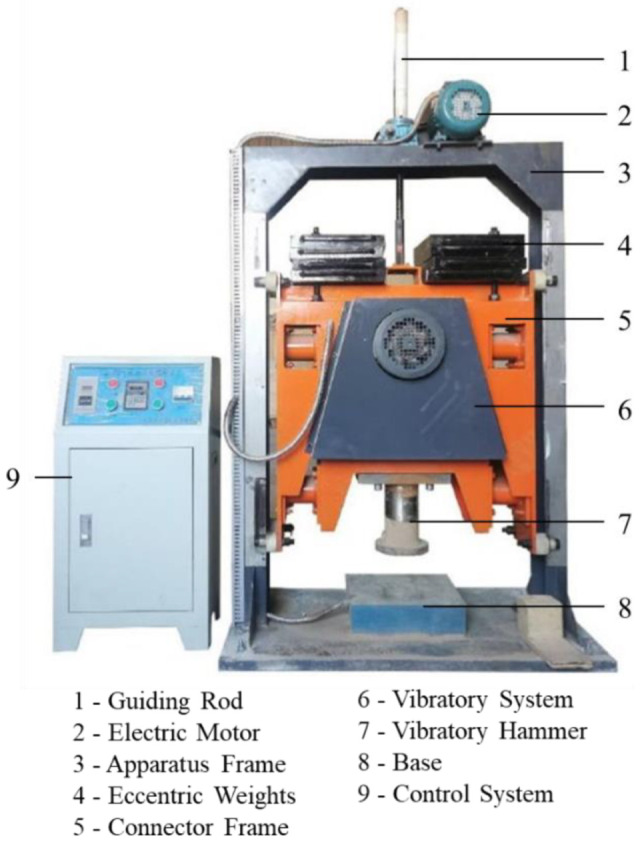
The new plate vibratory compaction apparatus developed at Central South University.

In this study, the laboratory vibratory plate compaction tests were conducted using an innovative apparatus developed at the Central South University. As shown in Figure 3, it mainly consists of static weights, the vibratory system, eccentric weights, and the control system. Vibratory compaction operates on the principle that an electric motor drives the eccentric vibratory weights. The two eccentric weights on the vibratory exciter have the same mass and rotation speed but in opposite directions. During the energy transmission process from the motor, the horizontal forces formed at the time cancel each other out, and the vertical component forces cause the indenter to vibrate vertically and compact the UPAB [32]. The vibratory compactor uses various eccentric weights combinations to change the magnitude of the excitation force and a frequency conversion controller to modify and vary the excitation frequency of the vibratory motor, which can achieve adjustment continuously in the frequency range of 1–35 Hz, thereby simulating the different actual working conditions of vibratory rollers.

### 2.3. Modified Proctor Compaction Test 

The initial moisture content of the samples with *G*/*S* values of 1.0, 1.6, 1.8, 2.0, 2.5, and 1.6 M, respectively, are 3%, 4%, 4.5%, 5%, and 6% in modified proctor compaction tests, where the inner diameter of the compacted sample is 152 mm, the height is 120 mm, the sample is compacted in three layers, with each layer subjected to 98 blows, resulting in compaction energy of 2677 kJ/m^3^.

**Figure 4:**
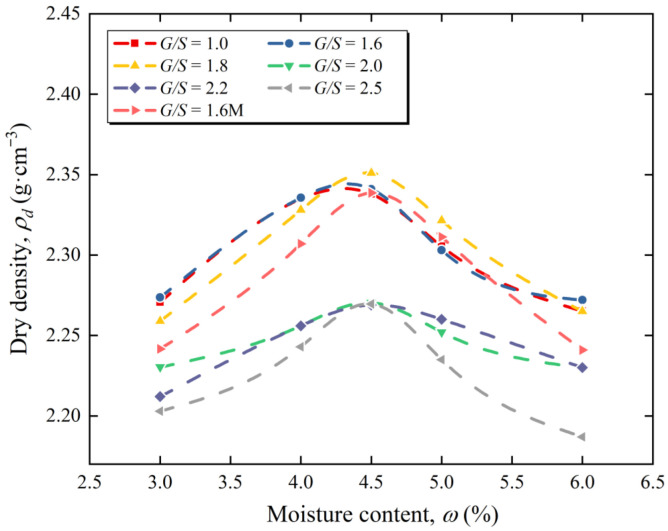
Proctor compaction curves of UPAB specimens with 7 designed gradations.

**Figure 5:**
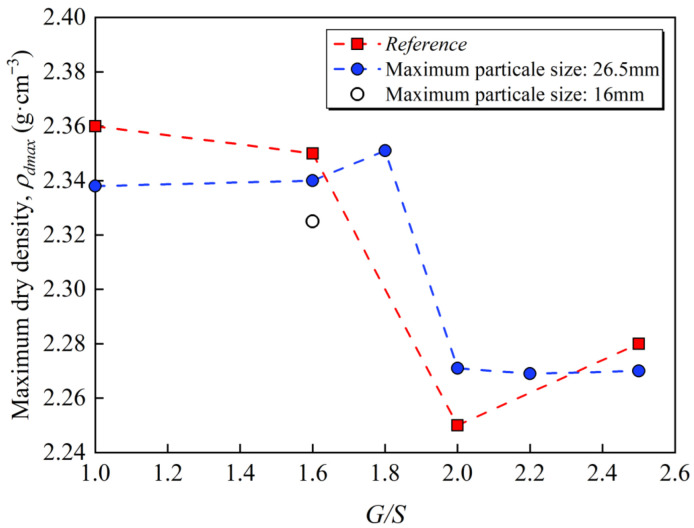
The achieved maximum dry density values were plotted against *G*/*S* values of UPAB specimens from proctor compaction tests.

Figure 4 shows modified proctor compaction curves of UPAB specimens with different gradations, and the moisture content and dry density curves of the UPAB specimens at 6 different *G*/*S* values can be obtained. Table 2 presents the corresponding maximum dry density and optimal moisture content in Figure 4. Accordingly, for various *G*/*S* levels, the compacted dry density of the UPAB firstly rises and then drops with the increase in moisture content, and each curve has a maximum dry density point that differs from the others. Figure 5 reveals that when the *G*/*S* value grows from 1.0 to 1.6, the proportion of fine particles in the UPAB specimen decreases significantly, while the maximum dry density increases slowly, indicating that the particle packing structure of the aggregates is still a floating dense type when the *G*/*S* value is between 1.0 and 1.6. As the *G*/*S* ratio rises from 1.6 to 1.8, the proportion of fine particles in the specimen slightly decreases. In contrast, the maximum dry density value exhibits a trend of increase, indicating that the particle packing structure of the materials has gradually transformed into a skeleton dense structure. In other words, the *G*/*S* value range between 1.6 and 1.8 corresponds to the skeleton dense structure type. Similarly, when the *G*/*S* value increases from 1.8 to 2.0, the content of fine particles in the specimens decreases slightly, but the maximum dry density drops significantly, demonstrating that the particle packing structure of the aggregates transforms into the skeleton void type. However, as the *G*/*S* value increases from 2.0 to 2.5, the proportion of fine particles in the specimen decreases significantly, whereas the maximum dry density almost has no change, showing that the original packing structure of the skeleton void type remains the same. On the other side, the gradation of *G*/*S* value of 1.8–2.5 belongs to the skeleton void structure type.

Chen [21] concluded that when the particle packing structure of unbound aggregates is skeleton void or floating dense type, its gradation can vary widely within a specific area of the skeleton dense type. It is inconsistent with the conclusion that the *G*/*S* value controls particle packing structure types of UPAB obtained by tests, namely the *G*/*S* = 1.0–1.6 grading for floating dense structure, *G*/*S* = 1.8–2.5 grading for skeleton void structure, and *G*/*S* = 1.6–1.8 grading for skeleton dense type structure, in which the floating suspended dense structure and skeleton void structure have a wide range. Thus, the gradation design of UPAB controlled by the *G*/*S* parameter can effectively distinguish three distinct particle packing structure, which has a specific control effect on the dry density of UPAB after compaction. The maximum dry density of the specimen after compaction initially grows and then drops with increasing the *G*/*S* value, and the optimal *G*/*S* = 1.8 can cause the dry density of the unbound aggregates to reach its maximum, which is consistent with the findings reported by Zheng et al. [29].

The particle packing structure of unbound aggregates is affected not only by gradation but also by the shape and size of aggregate particles. Accordingly, the aggregates’ maximum particle size and gradation shape parameters define the *G*/*S* value. Hence, the maximum particle size and gradation shape parameters of the aggregates with the same *G*/*S* are different. Two groups of specimens with G/S = 1.6 M (*G*/*S* = 1.6 and the maximum particle size for 16 mm) and *G*/*S* = 1.6 (*G*/*S* = 1.6 and the maximum particle size for 26.5 mm) were designed to compare the difference in compaction characteristics between UPAB with the same *G*/*S* but various particle maximum sizes. The comparison of the dry density after the proctor compaction test is depicted in Figure 5. It can be observed in this figure that the maximum dry density of specimens in *G*/*S* = 1.6 was higher than that in *G*/*S* = 1.6 M.

Yang [33] studied the effects of different gradations, particle shape characteristics, and maximum particle size on the compaction of sand and gravel materials using the field large-density cylinder method. They found that the maximum dry density of sand and gravel materials with different gradation shape parameters and maximum particle size could be different despite the same compaction method. The maximum dry density of sand and gravel material rises initially and then falls with the increase in the uniformity coefficient. As the curvature coefficient grows, the maximum dry density decreases gradually and tends to remain stable. When the maximum particle size is less than 100 mm, the maximum dry density rises as the maximum particle size grows. Unbound aggregates have similar characteristics compared to sand and gravel materials. From Table 1, *C_u_* > 5 and *C_c_* = [1, 3] when *G*/*S* = 1.6 and *G*/*S* = 1.6 M means the specimens are well graded. The uniformity coefficient of the specimen at *G*/*S* = 1.6 is larger than that at *G*/*S* = 1.6 M, but the curvature coefficient varies very little when the maximum particle size of the specimen is far less than 100 mm. After compaction, the specimens with the same *G*/*S* value but different maximum particle sizes have various maximum dry density values under the condition of proper gradation and utilizing the same compaction method, the maximum dry density increases as the uniformity coefficient and maximum particle size in a given range rise.

### 2.4. Plate Vibratory Compaction Tests

The specific plan of the plate vibration compaction test is detailed in Table 3. Six grades of UPAB samples with *G*/*S* values of 1.0, 1.6, 1.8, 2.0, 2.5, and 1.6 M were subjected to various exciting forces. The purpose of the plate vibration compaction test under the combination of vibration frequency and vibration time is to investigate the vibratory compaction characteristics of UPAB samples of different grades under different working conditions. The vibratory compaction sample has a 150 mm diameter. The sample is mixed with water based on the optimal moisture content obtained by the proctor compaction test, and it is placed in a tray sealed with plastic wrap for 24 h to ensure that the water and aggregates are thoroughly combined.

**Figure 6:**
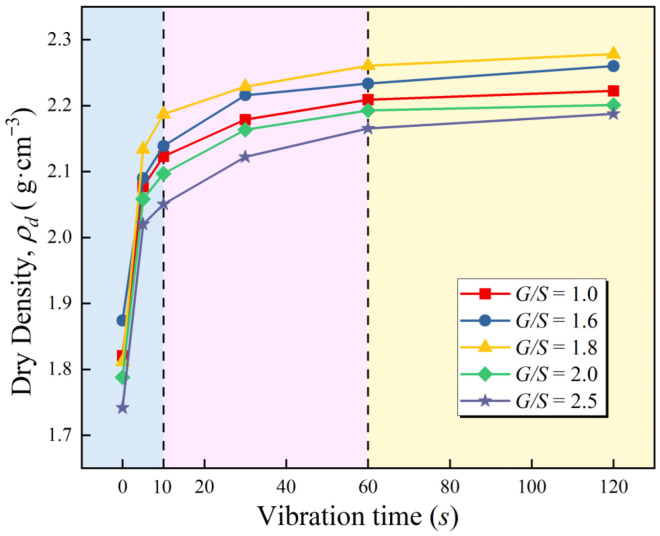
The curves of achieved dry density versus elapsed time from newly plate vibratory compaction tests.

The characteristics of dry density versus elapsed time curve at 5 *G*/*S* level of UPAB were similar. When the vibration frequency of the specimens was 25 Hz and the vibratory force was 1.4 kN, the dry density of the specimen grew with the increase in vibration time under the same conditions. In contrast, the dry density growth rate decreases and stabilizes as vibration time increases. Therefore, the whole vibration compaction process can be divided into three distinct stages, as illustrated in Figure 6: (1) the range of 0–10 s. In this stage, the shear stress generated by the vibratory force exceeds the friction force between particles, resulting in a rapid relative movement of aggregate particles under the action of the vibratory force, a decrease in the gap between particles under the action of external force, and a rapid enhancement of interlocking and occlusions between particles. (2) The range of 10–60 s. In this stage, the packing particles are constantly arranged closely, the friction between the particles gradually increases, the specimens form a strong skeleton, the resistance to deformation is improved, and the growth rate of the dry density is stabilized. (3) The range of 60–120 s. During this phase, the shear strength of the aggregates reaches parity with the external force. The shear stress produced by the vibratory force is insufficient to disturb the dense structure formed by the skeleton support force and the inter-particle friction resistance, and the aggregates reach the maximum compaction state under the action of the external force. As coarse particles can still be crushed by vibratory compaction, the crushed particles can further fill the void, resulting in a slight increase in the dry density value. However, a longer vibratory compaction time of unbound aggregates is not the best from an economic and efficiency perspective, and the optimal vibratory compaction time is between of 60 s to 80 s. 

**Figure 7:**
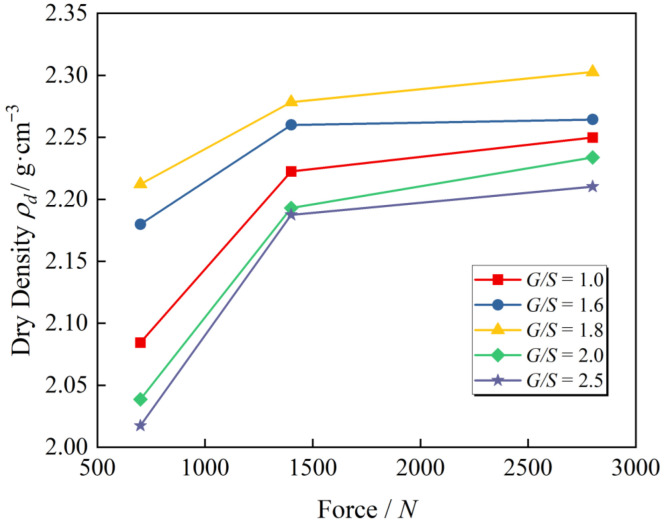
The curves of achieved dry density versus vibratory force from laboratory plate vibration compaction tests.

Figure 7 presents the curves of achieved dry density versus vibratory force from laboratory plate vibration compaction tests at an excitation frequency of 25 Hz. Although the gradation of the aggregate samples is different, the trends of dry density versus exciting force are comparable. In other words, when the exciting force is small, the dry density rises rapidly with the increase in vibratory force, whereas when the vibratory force exceeds a certain extent, the growth rate of dry density slows, and the compaction effect also declines. This is because the original equilibrium of internal force was broken by vibratory force. When the shear stress of particles surpasses the friction between particles, relative movement is generated. They are reorganized, arranged, and packed into a new skeleton structure, decreasing gaps and increasing densities. The larger the vibration load, the higher the energy required to fill the void created by the relative movement of particles, and the denser the aggregates. When the aggregate particles are compacted to a certain degree, the occlusion and compaction between particles increase to resist the disturbance of external vibratory force. Meanwhile, the external excitation is difficult to promote a denser packing state. Thus, the growth rate of dry density reduces, and particle breakage increases gradually. Therefore, an optimal vibration force maximizes the dry density of aggregate specimen after vibratory compaction.

**Figure 8:**
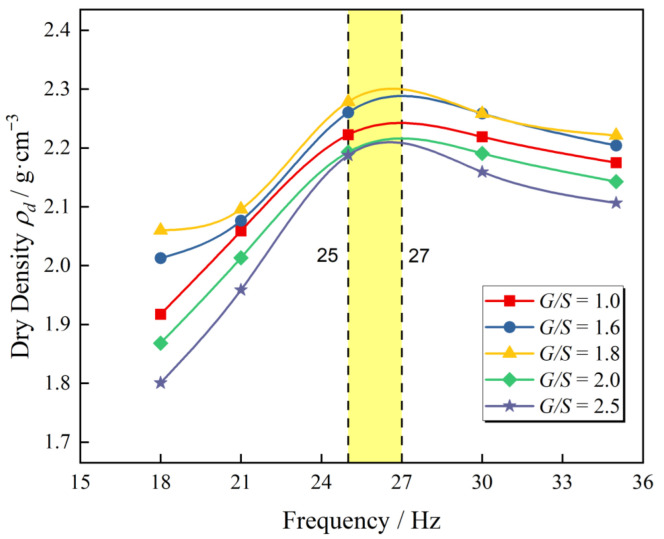
The curves of achieved dry density versus vibratory frequency from new plate vibratory compaction tests.

Figure 8 illustrates the curves of achieved dry density versus vibratory frequency at different *G*/*S* levels from laboratory plate vibration compaction tests with *F*_0_ = 1.4 kN and 120 s of compaction time. It can be observed that the dry density versus vibratory frequency curve of different gradations at various *F*_0_ levels approximately follows a normal distribution. On the other hand, under diverse vibratory force levels, an optimal frequency always exists (25–27 Hz), causing dry density to reach its maximum after vibratory compaction. The specimen under various *G*/*S* has different dry densities, but the optimal frequency corresponds to the same maximum dry density.

Researchers around the world proposed many theories on vibratory compaction mechanisms. A series of laboratory and field tests were conducted to verify the soil resonance theory [34]. The theory of resonant compaction is founded on the resonance principle in physics, in which resonance occurs when the frequency of the vibratory force reaches the range of the soil’s natural frequency. During this period, the displacement of the particles is at its largest, the friction between particles is significantly reduced, and the specimens are easy reach the compact state. Thus, compaction effectiveness is improved. Li [35] studied the variation of the natural frequency of subgrade soil under different degrees of compaction using the experimental modal analysis. It was discovered that the natural frequency of soil grows slowly with the increase in compaction levels. Up until a certain threshold, the natural frequency of soil shows a rapid increase. Dry density is a physical index representing soil’s degree of compaction and pore characteristics, while the degree of compaction reflects the compaction efficiency of different compaction methods. 

Table 4 lists the values of the degree of compaction of TRW-derived UPAB specimens with various *G/S* values under different vibratory conditions. It can be seen from Table 4 that under the application of the vibratory excitation force of *F*_0_ = 1.4 kN for instance, the variation ranges of the degree of compaction (i.e., the differences between the maximum and minimum ones) corresponding to the vibratory frequency levels of 18, 21, 25, 30, and 35 Hz were calculated as 8.3%, 2.86%, 1.85%, 1.61%, and 1.69%, respectively. Thus, greater variation range of the degree of compaction across different *G*/*S* values was observed for lower vibratory frequency. In other words, the compaction effectiveness of the UPAB specimens with different *G*/*S* levels was weaker when the excitation frequency was lower. With the increased vibratory frequency, the difference among the degree of compaction of specimens with different *G*/*S* values decreased rapidly. Interestingly, the achieved dry density values of specimens with different *G/S* values all reached their respective maximum values when the vibratory frequency was within 25–27 Hz. This may further support the above-mentioned resonance theory of soil compaction, i.e., such a frequency range of 25–27 Hz might be close to the natural frequency of UPAB materials with different *G/S* values, thus causing the resonance phenomenon and the densest packing due to compaction. 

As the vibration frequency continued to increase, the “decoupling” phenomenon started to appear between the vibratory compactor and the UPAB materials with different *G/S* values. This may be attributable to the sudden reduction of their respective achieved dry density and the increase in the variation range of the degree of compaction. For example, when the vibratory exciting force was *F_0_* = 2.8 kN, the variation range of the degree of compaction of UPAB specimens with different *G/S* values was calculated as 2.16%, 2.13%, and 1.92% under the vibratory frequency levels of 20, 25, and 29 Hz, respectively. It is obvious that the variation range of the degree of compaction of UPAB specimens with different *G/S* values decreased when the vibratory frequency reached the postulated natural frequency. Similar trends were also observed for *F*_0_ = 1.4 kN. For the sake of the operational safety of the vibratory plate compaction apparatus, high frequency levels of the vibratory force *F*_0_ = 2.8 kN were not attempted. By comparing the compaction quality of UPAB specimens with varying *G*/*S* values subjected to different combinations of vibratory force and frequency, it can be found that the vibratory force does not change the nature of UPAB materials. Thus, there seems to exist the optimal vibratory frequency of about 25–27 Hz that could potentially maximize the compaction effectiveness under different vibratory force levels.

**Figure 9:**
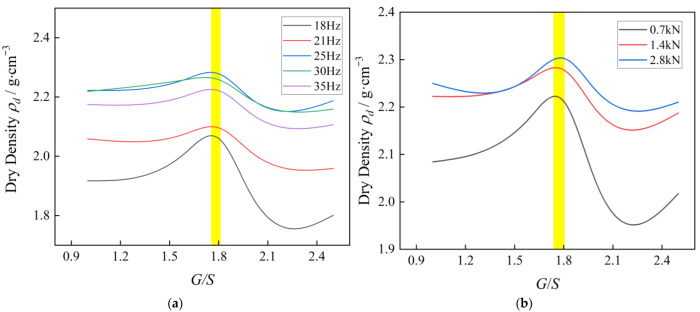
The curves of achieved dry density versus *G/S* value from laboratory plate vibratory compaction tests conducted at (**a**) fixed vibratory force (1.4 kN) but different vibratory frequency levels and at (**b**) fixed vibratory frequency (25 Hz) but different vibratory force levels.

Figure 9a shows achieved dry density curves of UPAB specimens varying with different *G/S* values under the application of vibratory excitation force *F_0_* = 1.4 kN with different frequency levels of *f* = 18, 21, 25, 30, and 35 Hz, respectively. It can be seen from Figure 9a that all the dry density curves gradually increase starting from *G/S* = 1.0 and eventually decrease after the *G/S* value reaches the peak of around 1.8. The variation ranges of the degree of compaction (i.e., the differences between the maximum and minimum ones) corresponding to the vibratory frequency levels of 18, 21, 25, 30, and 35 Hz were calculated as 8.3%, 2.86%, 1.85%, 1.61%, and 1.69%, respectively. As indicated by the results of such variation ranges of the degree of compaction, when the vibratory excitation frequency is close to the natural frequency of UPAB specimens, i.e., within the range of 25–30 Hz, the curves of achieved dry density versus *G/S* value exhibit indiscernible differences, thus making the gradation (or particle packing structure) quantified by the *G/S* value dominate the compaction quality. The same trends can be observed from Figure 9b as well, i.e., different *G/S* values (or different particle packing structures) of UPAB specimens show relatively similar influences on the achieved dry density under the combinations of 25-Hz vibratory excitation frequency and different vibratory force levels of 0.7, 1.4, and 2.8 kN. In summary, the influence of the *G/S* value on the achieved dry density under different combinations of vibratory excitation frequency and force gradually decrease and become stable upon the *G/S* value of around 1.8. This is consistent with the results of laboratory Proctor compaction tests.

**Figure 10:**
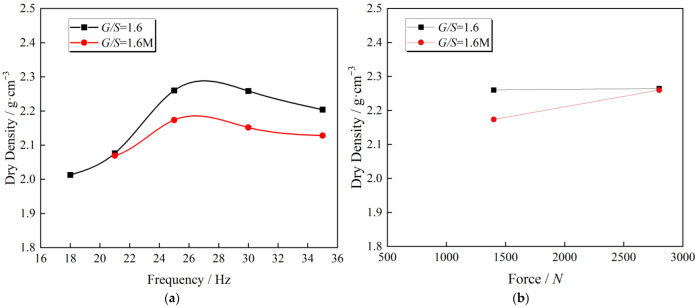
Comparison of achieved dry density after vibratory compaction between *G*/*S* = 1.6 and *G*/*S* = 1.6 M at (**a**) different vibratory frequency levels and at (**b**) different vibratory force levels.

Figure 10 presents the influence of maximum particle size on dry density after compaction under various vibration compaction conditions. Figure 10a illustrates the trends of dry density of aggregates with different *G*/*S* (*G*/*S* = 1.6 and *G*/*S* = 1.6 M) and maximum particle size under various vibratory frequencies when vibratory force *F*_0_ = 1.4 kN after 120 s compaction. With the increase of vibratory frequency from 18 Hz to 35 Hz, the dry density of packing samples with two different gradations exhibits a trend of first increase and then decrease, with the maximum dry density occurring around 25 Hz. In addition, the dry density of the aggregate specimens with *G*/*S* = 1.6 is higher than that with *G*/*S* = 1.6 M at each vibratory frequency. In other words, the larger the maximum particle size, the higher the dry density of the UPAB with the same *G*/*S* value after vibration compaction. Figure 10b represents the trends of dry density of aggregates with various *G*/*S* (1.6 and 1.6 M) and maximum particle size under different vibratory forces when vibratory frequency *f* = 25 Hz after 120 s compaction. The dry density of the two different specimens shows an increasing trend as the vibratory force rises from 700 N to 1400 N, but the dry density growth rate of the specimen with *G*/*S* = 1.6 is lower than that of the specimen with *G*/*S* = 1.6 M. The reason is that the maximum particle size of specimens with *G*/*S* = 1.6 M is smaller than that with *G*/*S* = 1.6, and the distribution of particles is uniform. The packing structure formed with lower vibratory force is more susceptible to destruction when subjected to higher vibratory force. For specimens with *G*/*S* = 1.6, their *C_c_* and *C_u_* are larger than specimens with *G*/*S* = 1.6 M. It is easier to form a denser packing structure, and it is more difficult to destroy by an increasing vibratory force. Hence, specimens with *G*/*S* = 1.6 M are more sensitive to the change in vibratory force than those with *G*/*S* = 1.6, and their dry density increases more rapidly as the vibratory force grows. The dry density of aggregate specimens with *G*/*S* = 1.6 under different vibratory forces is more significant than that with *G*/*S* = 1.6 M.

In conclusion, the proctor compaction or under the different vibration parameters combination of panel vibration compaction, the dry density of aggregate specimens with *G*/*S* = 1.6 after compaction is greater than that with the *G*/*S* = 1.6 M regardless of the compaction method used. Therefore, for the UPAB with the same *G*/*S*, their dry density is larger when the particle size, *C_c_* and *C_u_* are larger, particle size distribution is wider, and contact between coarse and fine aggregates is more uniform and has nothing to do with the compaction method.

## 3. Results and Analysis

### 3.1. Correlation Analysis of Different Plate Vibratory Compaction Variables

**Figure 11:**
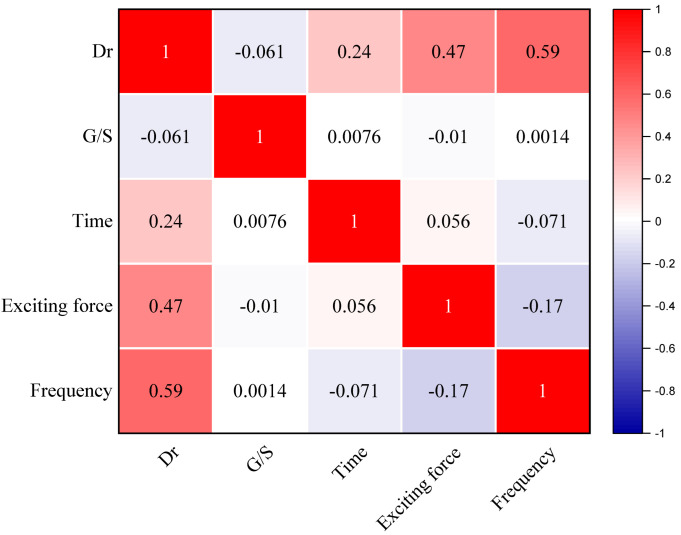
Correlation analysis between variables in plate vibratory compaction test.

The correlation analysis was conducted using the Pearson correlation method to investigate the influence of different variables, including gradation index, vibratory time, exciting force, and vibratory frequency for a degree of compaction in the plate vibratory compaction test. Figure 11 shows that the exciting force and frequency are −0.061, 0.24, 0.47, and 0.59, indicating that time, exciting force, and frequency positively correlate with the degree of compaction, whereas the gradation index *G*/*S* value correlates negatively with it. Moreover, the vibratory frequency has a much higher impact than other parameters. Therefore, it is crucial to control the priority of variables when designing the field of lab vibratory compaction tests or construction.

### 3.2. Effects of Different Compaction Methods on the Maximum Dry Density

**Figure 12:**
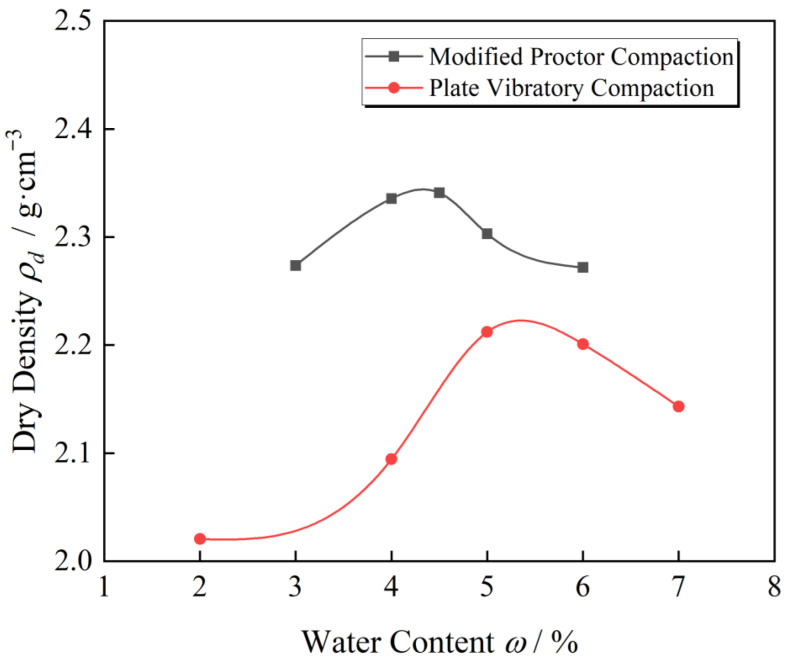
Compaction curves of UPAB specimens with *G*/*S* = 1.6 from different laboratory compaction methods.

Both compaction tests were performed on aggregates with *G*/*S* = 1.6 to compare the discrepancy between proctor compaction and plate vibration compaction with regard to compaction characteristics of UPAB. The modified proctor compaction test was referred to as ASTMD1577-12, while the plate vibratory compaction test was conducted using the optimal parameter combination obtained above (vibratory frequency *f* = 25 Hz, vibratory force *F*_0_ = 1.4 kN, and vibratory compaction time *t* = 120 s). Table 5 presents the moisture content of specimen and dry density results after two compaction methods, while Figure 12 illustrates the compaction curves of UPAB specimens with a *G*/*S* value of 1.6 generated from various laboratory compaction methods. 

Figure 12 indicates that regardless of the compaction method used, the dry density of aggregate specimens with *G*/*S* = 1.6 first rises and then drops with increasing moisture content. The maximum dry density of the aggregate specimens with *G*/*S* = 1.6 obtained from impact compaction is greater than vibratory compaction, whereas the optimal moisture content of the specimens is the opposite. Guo [34] described that the maximum dry density of coarse granular soil grew with the compaction work while the optimal moisture content decreased. The internal mechanism of the changing trends of optimal moisture content and maximum dry density can be explained by calculating the compaction work exerted on specimens by the two distinct compaction methods. Compared to the method outlined in the specification, the impact compaction technique calculates the work done to the specimens by each compaction based on the weight and drop distance of the heavy hammer. The heavy hammer exerts 5.83 kJ of compaction force on each specimen during the entire compaction process. For plate vibratory compaction, vibratory intensity, namely the vibration energy output per unit time, can be utilized to represent the compaction ability [35]. The corresponding calculation is shown in Equations (3) and (4).

(3) $$E_{1}=2Af\left(Wg+\frac{\pi F_{0}}{4}\right)=fE_{0},$$

(4) $$A=\frac{m_{e}}{W},$$

where *E*_1_ is the vibration energy (J) output per second by the plate vibratory compactor, *E*_0_ is the vibratory energy (J) output by the plate vibratory compaction instrument in each cycle, *f* is the excitation frequency (25 Hz), *W* is the dead weight of the plate vibratory hammer (330 kg), *g* is the acceleration of gravity (9.8 m·s^−2^), *F*_0_ is the vibration force (1.4 kN), *m_e_* is the static eccentricity of the eccentric mass block (0.057 m), *A* is the nominal amplitude (m).

According to Equation (3), the total energy of the plate vibratory compaction in 120 s was *E*_1_ = 4.47 kJ, which was less than the total energy of modified proctor compaction (5.83 kJ). Therefore, under the optimal vibratory parameter combination with *G*/*S* = 1.6, the maximum dry density obtained from vibratory compaction was lower than impact compaction, while the optimal moisture content is the opposite consistent with Guo’s findings [34]. In contrast, the steel compaction head of the plate vibratory compaction instrument has a significant action area with the aggregates, resulting in the compression settlement of aggregates. Meanwhile, the friction resistance of the side wall to the aggregates is relatively high, while the influence of side wall friction is relatively low in impact compaction because the heavy hammer diameter is smaller than the compaction cylinder one. In the impact compaction test, the compaction effect of load in compaction is superior to that of vibratory compaction due to the free fall of the compaction hammer, the pressure generated by the local impact force is greater than that of vibratory compaction, and the impact energy transfer depth is better than that of vibratory compaction, which is also consistent with the results of [36,37]. Thus, when the output energy of vibratory compaction is less than or close to that of impact compaction, the maximum dry density obtained by impact compaction is higher than vibratory compaction, while the optimal moisture content is the opposite.

### 3.3. Influence of Different Compaction Methods on Particle Breakage

Plate vibration and modified proctor compaction have a direct impact force on the crushed stone packing. The test process is likely to break the graded crushed stone packing particles, resulting in a large deviation in the actual gradation of the packing after the packing is compacted, which significantly impacts the performance of coarse-grained soil fillers. Marsal proposed that the particle crushing rate is the sum of the absolute value of the difference between the particle size content before and after the test representing the change in the gradation throughout the crushing process [38]. The larger the value, the more significant the crushing phenomenon. In order to quantitatively analyze the influence of the two compaction methods on the gradation of graded crushed rock before and after compaction, the crushed rock filler after the proctor compaction and vibration compaction tests is washed and screened, and proctor compaction and vibration under various working conditions are calculated. After compaction, the crushing rate of particles with a particle size of at least 2.36 mm.

**Figure 13:**
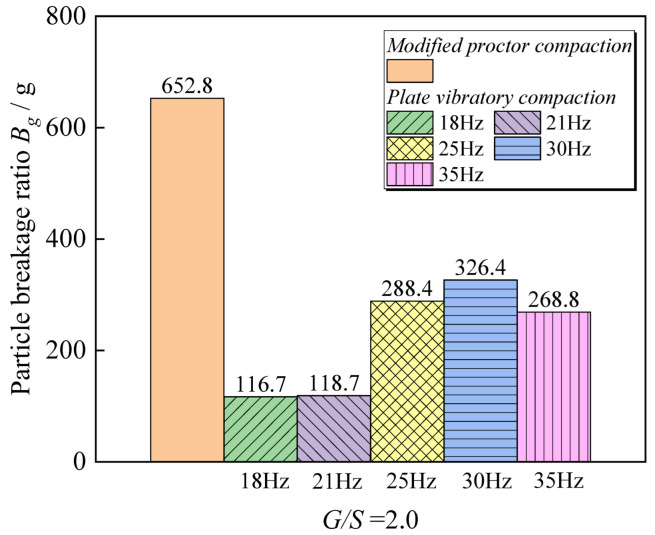
Particle breakage *Bg* values for UPAB specimens with a *G*/*S* value of 2.5 under different combinations of vibratory parameters.

Figure 13 shows the statistical results of the particle crushing rate of the graded crushed stone packing with *G*/*S* = 2.5 subjected to different vibration compaction conditions. It reveals that both modified proctor and plate vibratory compaction will change the level. With the gradation of crushed stone packing, the particle crushing rate value under modified proctor compaction is greater than that of vibratory compaction due to the drop of the hammer in proctor compaction. In the vibration compaction test, the height exceeds the vibration amplitude by a wide margin. The pressure generated by the local impact force of the compaction hammer is greater than that of the vibration compaction, and the impact energy transmission depth is higher, which also causes the coarse particles in the graded crushed stone packing to be more likely broken. Under the plate vibratory compaction, when the excitation force is the same for the graded crushed stone packing of various types of structures, the particle breakage rate shows a trend of increasing and then decreasing as the frequency rises, excitation frequency is close to that of the soil. The phenomenon of particle fragmentation is the most significant when the natural frequency is in the body.

**Figure 14:**
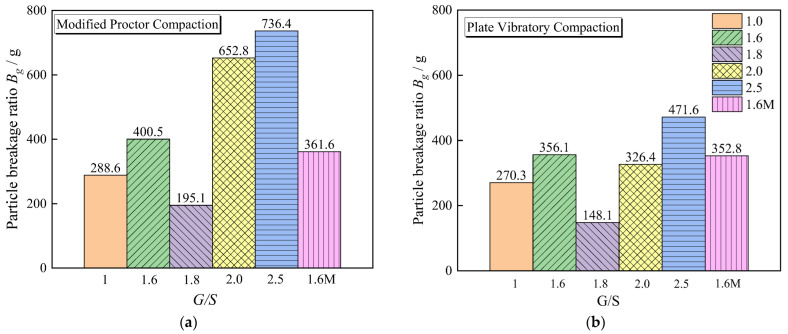
Particle breakage Bg values for UPAB specimens with different *G*/*S* levels under: (**a**) Modified proctor compaction; (**b**) Plate vibratory compaction with 30 Hz frequency.

Figure 14 exhibits the statistical results of the particle breakage rate of aggregate samples with different *G*/*S* values after modified proctor compaction and plate vibratory compaction with an excitation frequency of 30 Hz. The smallest value of *Bg* occurs when the *G*/*S* value is near 1.8 for both compaction methods, indicating that the dense structure of the skeleton has not only the close contact of large particles to form a stressed skeleton but also corresponding small particles filling the voids of the skeleton, resulting in higher shear strength and deformation resistance under the action of external force. The particles are evenly matched and resistant to breakage. Comparing the particle breakage of the filler samples with *G*/*S* = 1.6 and *G*/*S* = 1.6 M under two distinct compaction methods reveals that the filler samples with *G*/*S* = 1.6 M are compacted using the same method. The sample with a particle crushing rate value less than *G*/*S* = 1.6 indicates that the larger the maximum particle size of the water-permeable graded crushed stone filler, the more significant the particle crushing.

### 3.4. CT Scanning Analysis

**Figure 15:**
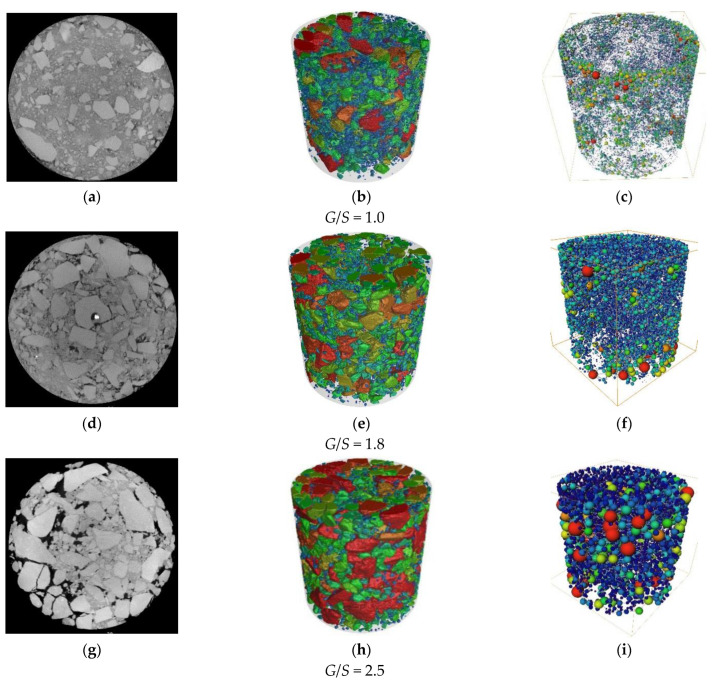
Representative XCT slices (**a**,**d**,**g**), reconstructed 3D model (**b**,**e**,**h**), and the equivalent Ball-Beam model (**c**,**f**,**i**) of UPAB specimens with different *G*/*S* levels (1.0, 1.8, and 2.5) during vibratory compaction from the left to the right for each row, respectively.

After the 60 s of plate vibration, the aggregate samples with *G*/*S* values of 1.0, 1.8, and 2.5 were subjected to a CT scan. Figure 15 depicts the representative CT slices of the above three different particle accumulation structure types (the three-dimensional weight of the sample). The structure result shown in Figure 15, Figure 15c is a three-dimensional void ball and stick model. The “ball” is a sphere with the same volume as the void, and the “stick” is the channel connecting the void to the void, whose size is determined by the size of the ball. The void volume distribution and the connectivity between the voids can be evaluated based on the number of sticks. It can be seen that the *G*/*S* = 1.0 sample has limited mutual contact between the coarse particles, and most of the coarse particles are “scattered” in the fine particles to form a dense suspension Structure, and the sample gap is small. The sample with *G*/*S* = 1.8 has coarse particles in contact with each other to form a skeleton, and the fine particles fill the gaps of the coarse particles well. The sample with *G*/*S* = 2.5 has the highest content of coarse particles with large voids. The area marked by the red circle in Figure 15a illustrates the particle fragmentation phenomenon in the sample. It is worth mentioning that the bright spot on the particle at the center of the CT slice of the sample with *G*/*S* = 1.8 is the steel inserted in advance. Nails are utilized to mark the movement trajectory of particles.

**Figure 16:**
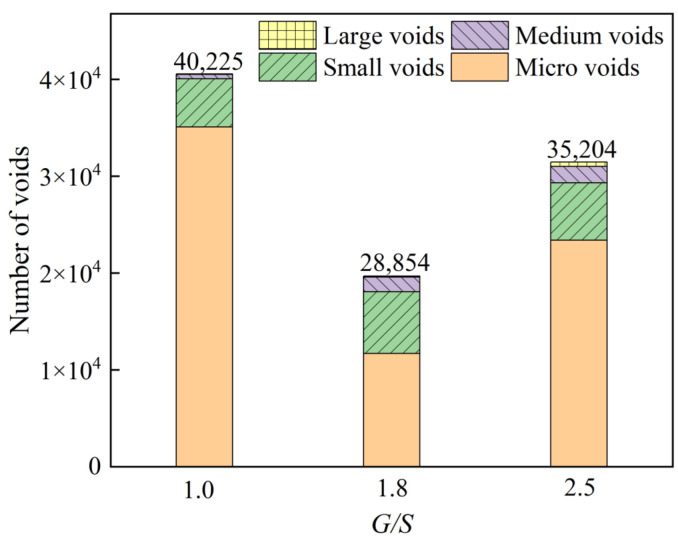
The number of voids of varying volumes counted for UPAB specimens with different *G/S* levels compacted by plate vibratory compaction.

Huining [39] studied the morphological differences of voids at various scales in asphalt mixtures and categorized the voids into different sizes according to their volume: larger than 100 mm^3^ for large voids, 20–100 mm^3^ for medium voids, and 5–20 mm^3^ for small voids Gaps and less than 5 mm^3^ are micro-voids. Accordingly, this paper counts the number of different void volumes in various compaction stages, as shown in Figure 16. It can be seen from the figure that the internal voids of the filler samples with different *G*/*S* values (different particle accumulation structure types) after vibration compaction are mainly micro-voids. Combining the ball and stick models depicted in Figure 15c, the sample with *G*/*S* = 1.0 has many fine particles in the suspended compact structure. The coarse particles do not contact to form a skeleton structure, and the fine particles play no role in the filling process. The number (balls) is the largest, mainly isolated micro-voids and small voids between fine particles and part of coarse particles. The fine particles in the dense skeleton structure of the sample with *G*/*S* = 1.8 can fill the skeleton voids formed between the coarse particles, so compared to the suspension denseness of *G*/*S* = 1.0 and the skeleton void structure of *G*/*S* = 2.5, The number of internal voids is significantly reduced, and the dry density of the sample after compaction is also the greatest. The number of fine particles in the void structure of the framework with *G*/*S* = 2.5 is small. Although the framework can be formed, the residual voids are large, especially when comparing the number of medium and large voids at *G*/*S* = 1.0 and *G*/*S* = 1.8. The growth is evident, the connectivity between the voids is optimal, the total number of voids is also moderate, and the dry density after compaction is the lowest.

### 3.5. Saturation-Based Unified Compaction Curve

The main purpose of the laboratory compaction test is to determine the appropriate moisture content for the on-site filling and rolling construction so that the soil can economically and effectively reach a well-compacted state. The traditional laboratory compaction test obtains the relationship curve between the moisture content and the dry density after compaction by applying the work corresponding to the field compaction on the soil. The compaction curve is typically used to determine the optimal moisture content required to control the field rolling construction and calculate the compactness index used to evaluate the on-site compaction effect according to the maximum dry density value corresponding to the optimal moisture content. In the construction of subgrade on-site filling and rolling, higher compaction energy is usually used to increase the soil’s post-compaction dry density, thus improving the soil’s strength and modulus while reducing the permanent deformation under the action of traffic load cycles, decreasing later operation and maintenance costs. However, this easily reduces the compaction energy applied by the indoor compaction compared to the on-site one, and the compaction degree often exceeds one, which requires using the moisture content-dry density curve to evaluate the compaction. The effect of the method and the on-site rolling construction are relatively low. Nevertheless, the development of compaction theory and technology allowed some scholars to suggest using the stiffness of the compacted soil (such as deflection test, foundation coefficient K30, etc.) for controlling the compaction quality. However, a study by Fumio Tasuoka [40] concluded that for the same soil type at a fixed moisture content, the stiffness of the soil does not increase monotonically with the increase in the soil’s dry density upon compaction. As a result, the method of controlling the compaction quality by the stiffness of the soil also has certain limitations. On the other hand, for the target dry density value, the stiffness of the soil increases significantly with the increase in saturation, and the compaction-saturation normalized curve obtained by using saturation instead of water content is different for different compaction energy levels of the same soil, making it insensitive enough for practical applications. Accordingly, this article explores the method’s applicability to compare and evaluate the compaction characteristics of the same material with different soil grading under different compaction methods and energies.

**Figure 17:**
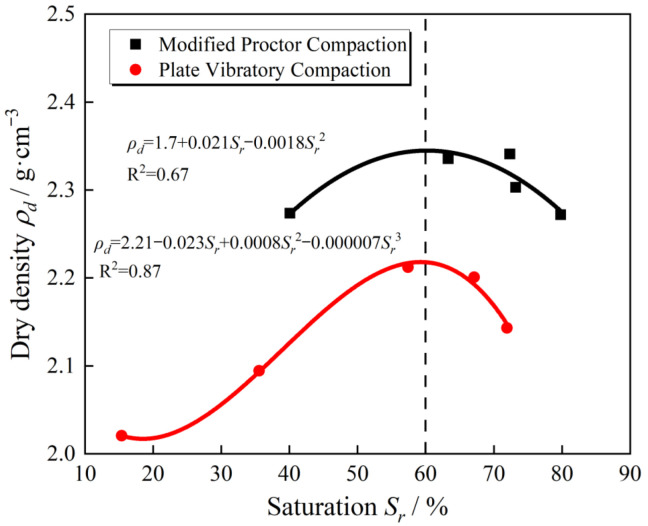
The curves of achieved dry density versus degree of saturation of UPAB specimens with a *G/S* value of 1.6 as generated from different laboratory compaction methods.

Figure 17 shows the saturation-dry density relationship curve of the filler sample with *G*/*S* = 1.6 under two different compaction methods. The water-permeable crushed stone filler of the same gradation exists under different compaction methods and energies. Almost the same optimal saturation (0.6) maximizes the dry density after compaction. Figure 17 shows the saturation-dry density relationship curve obtained by modified proctor compaction for UPAD with different *G*/*S* values. Indeed, different UPAB gradations are under the same compaction method and energies. Nevertheless, there is a difference in the optimal saturation, so it is necessary to normalize the saturation-dry density relationship curve.

**Figure 18:**
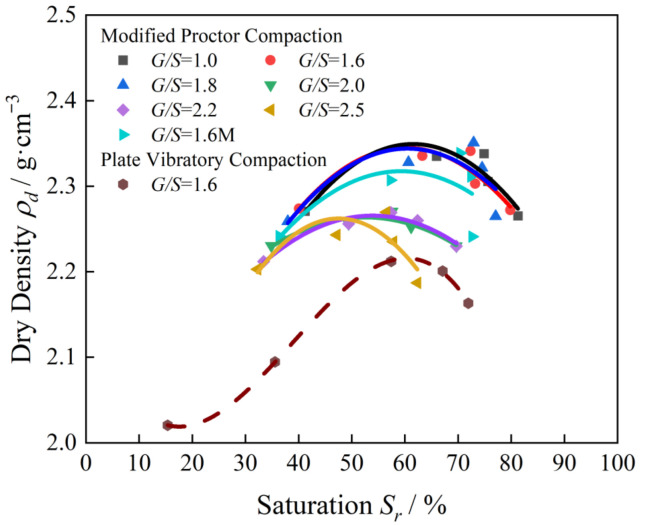
The curves of achieved dry density versus degree of saturation of UPAB specimens with different G/S values.

**Figure 19:**
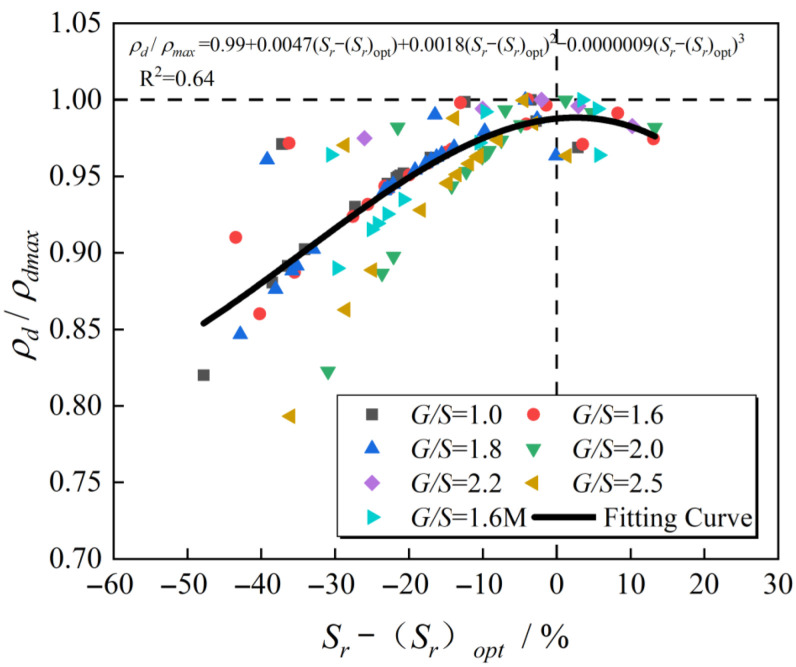
The unified compaction curve of UPAB specimens.

The dry density ratio after plate vibration compaction to the corresponding proctor compaction maximum dry density of the same grading packing sample under different combinations of excitation frequency and excitation force is taken as the ordinate. The packing sample after plate vibration compaction saturation of the corresponding proctor compaction optimal saturation (corresponding to the optimal moisture content of the proctor compaction) is used as the abscissa to obtain the saturation of the water-permeable crushed stone filler samples with different gradations as shown in Figure 18, which is the relationship curve between the different values and the dry density ratio (or degree of compaction). In this paper, the fitted curve (Figure 19) is referred to as the “dry density-saturation normalized compaction curve”. The optimal saturation corresponding to the maximum dry density of the filler samples of the same gradation under different compaction methods and energy is identical, and the water-permeable graded crushed stone filler samples with varying *G*/*S* values are compacted using a variety of methods. The relationship between dry density and water content under the action of compaction energy and the dry density-saturation normalization process shows a trend of first increasing and then decreasing. Compared to the traditional dry density-water content compaction curve, the normalized compaction curve is not sensitive to changes in compaction energy and aggregate type.

Given the field subgrade construction process, the aggregate type and compaction energy will often fluctuate to a certain extent with the progress of the compaction operation, such as the gradation change before and after compaction, the parameter change of the vibratory roller, etc. Therefore, the dry density-moisture content curve obtained by the traditional laboratory compaction test is more sensitive to changes in filler type and compaction energy, and it is difficult to guide and evaluate the on-site compaction effect accurately and the above-mentioned relation of dry density vs. degree of saturation. The normalized compaction curve can effectively combine the laboratory compaction test and the on-site rolling construction without considering the compaction energy and unknown changes in the type of filler particle accumulation structure to calculate the roadbed rolling at different times accurately. The degree of compaction achieved by the working layer. The specific steps are as follows: (1) Measure the dry density-moisture content curve of the filler through an indoor impact test (such as vibration compaction or proctor compaction, etc.) to obtain the maximum dry density and its corresponding optimum saturation. Based on this, calculate the degree of compaction and saturation under each moisture content and obtain the above-mentioned dry density-saturation normalized compaction curve; (2) Measure or estimate the saturation of the roller compacted soil on site according to the dry density-saturation normalized compaction curve to get the true compaction K of the layer in the current state. In addition, when the saturation and dry density of the on-site roller compacted soil is accurately obtained, the actual maximum dry density of the soil under the on-site roller compaction construction conditions can be measured, which can then be used to evaluate different specifications of road rollers and their rolling technology. Different compaction methods and energy provided by the parameters affect the compaction effect and quality of the subgrade rolling soil layer.

## 4. Summary and Conclusions

In this paper, the compaction characteristics of tunnelling rock wastes (TRW) recycled for use as unbound permeable aggregate base (UPAB) materials were studied from conventional laboratory Proctor compaction and newly introduced vibratory plate compaction tests. A total of six different permeable gradations were designed by the gravel-to-sand ratio (*G/S*) concept of the particle packing theory. The effects of gradation and compaction methods on the compaction characteristics of TRW-derived UPAB materials and the optimal combination of vibratory parameters were investigated. The normalized compaction curves of degree of saturation versus achieved dry density were found insensitive to variations in material gradations, compaction methods, and compaction energy levels, thus justifying its use for vibratory compaction quality evaluation and control. The major conclusions were derived from the findings as follows:

(1) The gradation design method controlled by the gravel-to-sand ratio can effectively divide the gradation of UPAB materials into three different particle packing structures defined as suspended dense, skeleton dense, and skeleton void for *G*/*S* ranges of 1.0 to 1.6, 1.6 to 1.8, and 1.8 to 2.5, respectively. The *G*/*S* parameters have a significant control effect on the post-compaction dry density of UPAB materials. In addition, the maximum achieved dry density increases to a *G*/*S* of 1.8 and then decreases with the increase in the *G*/*S* value.
(2) The maximum dry density values of the UPAB specimens at constant *G*/*S* value and different maximum particle sizes are also different. The larger the values of coefficient of uniformity and maximum particle size in a specific range under good gradation, the higher the maximum dry density values obtained by the same compaction method.
(3) The achieved dry density of UPAB materials during three different stages changes with time under plate vibratory compaction. From the perspective of economy and efficiency, the optimal time of plate vibratory compaction is between 60 s and 80 s, and the optimal frequency under different excitation forces is 25 to 27 Hz, which helps the specimens’ post-compaction dry density to reach its peak.
(4) At small output energy levels of vibratory plate compaction or cases with energy level close to that of the Proctor compaction, the maximum dry density of UPAB specimens with the same gradation under proctor compaction is greater than that of vibratory plate vibration. Furthermore, the optimal moisture content is lower in the case of Proctor compaction.
(5) Compared with vibratory compaction, the Proctor compaction has a more significant impact on the gradation change of UPAB materials before and after compaction, and the particle breakage is more serious. The TRW-derived UPAB materials with a *G*/*S* value of 1.8 has the lowest crushing rate and the highest dry density.
(6) The internal pores in TRW-derived UPAB materials with different *G*/*S* values after vibratory plate compaction are mainly micro-pores, and the specimens with a *G*/*S* value of 1.8 have the least number of pores and the closest arrangement of particles after vibratory compaction, thereby increasing the dry density.
(7) The unified (or normalized) compaction curve of achieved dry density versus degree of saturation is relatively insensitive to the changes of material gradations, compaction methods, and compaction energy levels. Thus, compared with the conventional compaction curves, this method can accurately evaluate and control the compaction quality of field construction of TRW-derived UPAB layers.

## Figures and Tables

**Table 1:** Gradation design scheme and corresponding gradation parameters for Laboratory Compaction Tests.

*G/S*	*D*_max_ (mm)	*n*	*C_u_*	*C_c_*
1.0	26.5	0.32	270.22	3.55
1.6	26.5	0.51	29.09	1.97
1.8	26.5	0.56	23.64	2.08
2.0	26.5	0.61	18.09	1.90
2.5	26.5	0.71	11.63	1.63
1.6 M	16	0.77	9.84	1.63

**Table 2:** Results of laboratory proctor compaction test.

*G/S*	Maximum Dry Density (g·cm^−3^)	Optimum Moisture Content (%)
1.0	2.338	4.710
1.6	2.340	4.760
1.8	2.351	4.762
2.0	2.271	4.400
2.2	2.269	4.660
2.5	2.270	4.850
1.6 M	2.325	4.450

**Table 3:** Testing matrix of laboratory plate vibratory compaction tests.

*G/S*	Vibration Force (kN)	Vibration Frequency (Hz)	Vibration Time (s)
1.0/1.6/1.8/2.0/2.5	0.7	25	0/5/10/30/60/120
1.0/1.6/1.8/2.0/2.5/1.6 M	1.4	18/21/25/30/35	
1.0/1.6/1.8/2.0/2.5	2.8	20/25/29	

**Table 4:** The degree of compaction K of UPAB specimens with different *G*/*S* values.

*G/S*	*F_o_* = 0.7 kN *K*/(%)	*F_o_* = 1.4 kN *K*/(%)					*F_o_* = 2.8 kN *K*/(%)		
	25 Hz	18 Hz	21 Hz	25 Hz	30 Hz	35 Hz	20 Hz	25 Hz	29 Hz
1.0	89.15	82.01	88.06	95.06	94.90	93.02	94.52	96.23	95.19
1.6	93.16	86.02	88.73	96.58	96.51	94.19	95.09	96.77	95.86
1.8	94.10	87.63	89.15	96.91	96.06	94.48	95.85	97.94	96.46
2.0	89.77	82.26	88.66	96.56	96.46	94.36	96.68	98.36	96.29
2.5	88.87	79.33	86.29	96.37	95.12	92.79	96.28	97.37	94.54
1.6 M	NA	NA	89.01	93.49	92.54	91.52	NA	97.20	NA

**Table 5:** Compaction results of UPAB specimens with *G*/*S* = 1.6 from different laboratory compaction methods.

**Modified Proctor compaction**	Moisture content/(%)	3	4	4.5	5	6
	Dry density/(g·cm^−3^)	2.27	2.34	2.34	2.30	2.27
	Optimum moisture content/(%)	5.03	5.03	5.03	5.03	5.03
	Maximum dry density/(g·cm^−3^)	2.22	2.22	2.22	2.22	2.22
**Vibratory plate compaction**	Moisture content/%	2	4	5	6	7
	Dry density/(g·cm^−3^)	2.12	2.20	2.33	2.32	2.26
	Optimum moisture content/(%)	5.03	5.03	5.03	5.03	5.03
	Maximum dry density/(g·cm^−3^)	2.22	2.22	2.22	2.22	2.22

## Data Availability

Some or all data, models, or codes that support the findings of this study are available from the corresponding author upon reasonable request.
